# Phase I trial combining gemcitabine and treosulfan in advanced cutaneous and uveal melanoma patients

**DOI:** 10.1038/sj.bjc.6602586

**Published:** 2005-05-10

**Authors:** P G Corrie, J Shaw, V J Spanswick, R Sehmbi, A Jonson, A Mayer, R Bulusu, J A Hartley, I A Cree

**Affiliations:** 1 Oncology Centre, Addenbrooke's Hospital, Cambridge CB2 2QQ, UK; 2 Cancer Research UK Drug-DNA Interactions Group, Department of Oncology, Royal Free and University College Medical School, London W1W 7BS, UK; 3 Department of Oncology, Royal Free Hospital, London NW3 2QG, UK; 4 Translational Oncology Research Centre, Queen Alexandra Hospital, Portsmouth PO6 3LY, UK

## Abstract

Gemcitabine and treosulfan are DNA-damaging agents. Preclinical studies suggest that synergism exists when melanoma cells are exposed to both drugs concurrently. We conducted a phase I trial in advanced melanoma patients to determine the optimal dose of gemcitabine to be combined with treosulfan. Cohorts of three patients received increasing doses of gemcitabine, commencing at 0.5 g m^−2^, followed by a fixed dose of 5.0 g m^−2^ treosulfan on day one of a 21-day cycle. Patients alternately received a first cycle of single-agent gemcitabine or treosulfan before subsequent cycles of both drugs. Peripheral blood lymphocytes were collected in cycles 1 and 2 at various time points until 48 h post-treatment. The single-cell gel electrophoresis (Comet) assay was used to measure chemotherapy-induced DNA damage. A total of 27 patients were enrolled, no objective responses were observed, but two uveal melanoma patients had minor responses. Dose-limiting myelosuppression was reached at 3.0 g m^−2^ gemcitabine. DNA single-strand breaks were detected 4 h post-gemcitabine, repaired by 24 h. DNA interstrand crosslinks were detected 4 h post-treosulfan, fully removed by 48 h. Following combination chemotherapy, treosulfan-induced DNA crosslinks persisted, still being detectable 48 h post-treatment, supporting the hypothesis that gemcitabine potentiates treosulfan-induced cytotoxicity. The recommended regimen for further study is 2.5 g m^−2^ gemcitabine combined with 5.0 g m^−2^ treosulfan.

Metastatic melanoma is a devastating disease. Melanomas most commonly arise from the skin, more rarely from the uveal tract in the eye. The incidence of melanoma varies worldwide. In western countries, it stands at around 10–50 per 100 000 per annum, and its incidence is increasing faster than any other type of cancer. Uveal melanoma, although the most common primary malignancy involving the eye, is extremely rare, with annual incidence being around seven per million of the population. On biological and molecular grounds (Prescher *et al*, 1994; Woll *et al*, 1999; Davies *et al*, 2002; Kefford *et al*, 2004), uveal melanoma is a separate disease distinct from cutaneous melanoma. However, if local control fails, prognosis for both groups of patients is extremely poor, with median survival time being around 6 months (Woll *et al*, 1999; Chang *et al*, 1998; Balch *et al*, 2001). Melanoma is highly resistant to chemotherapy and immunotherapy and no systemic therapy has yet been shown to impact on survival of either patient group (Bedikian *et al*, 1995; Nathan *et al*, 1997; Tsao *et al*, 2004).

Recently, the novel chemotherapy combination regimen, gemcitabine plus treosulfan (gemtreo), has shown preclinical (Cree *et al*, 1999; Neale *et al*, 1999) and clinical (Mayer *et al*, 1999; Szelenyi *et al*, 1999; Pfohler *et al*, 2003) evidence of activity in both cutaneous and uveal melanoma. Studies suggest that synergism may exist between the alkylating agent, treosulfan and the nucleoside analogue, gemcitabine, currently used to treat several chemoresistant cancers. Gemcitabine is incorporated into DNA which leads to masked chain termination, the major direct cytotoxic action. It also inhibits ribonucleotide reductase, the enzyme that produces deoxynucleotides required for DNA synthesis and repair. As such, it is an agent which lends itself to combination strategies, with potential to modulate cytotoxicity of other drugs such as alkylating agents.

Treosulfan is a prodrug and is converted nonenzymatically to L-diepoxybutane under physiological conditions (Feit *et al*, 1970). Following conversion, alkylation of DNA and subsequent formation of DNA interstrand crosslinks is considered to be the mechanism by which treosulfan elicits its cytotoxicity (Hartley *et al*, 1999a, 1999b).

The optimal administration schedule of gemcitabine and treosulfan is not known, but clinical and pharmacological evidence suggest that the two drugs are best administered on a single day.

A phase I study was undertaken to determine (1) the maximum tolerated dose of gemcitabine combined with a fixed dose of treosulfan, administered on day 1 of a 21-day cycle, (2) safety, toxicity and efficacy of the combination chemotherapy regimen and (3) whether gemcitabine affects the persistence of treosulfan-induced DNA interstrand crosslinks.

## PATIENTS AND METHODS

### Patients

Patients with histological or cytological diagnosis of unresectable, metastatic cutaneous or uveal malignant melanoma and aged 18 years or more were eligible for this study. Patients were required to have bidimensionally measurable lesions, by either clinical or radiological methods; predicted life expectancy of 12 weeks or more and Eastern Cooperative Oncology Group (ECOG) performance status ⩽2. One line of prior chemotherapy was allowed, in which case at least 6 weeks must have elapsed since administration of previous treatment. Pretreatment laboratory requirements were as follows: haemoglobin ⩾10 g dl^−1^, platelets ⩾100 × 10^9^ l^−1^, ANC ⩾1.5 × 10^9^ l^−1^, total bilirubin <1.3 × upper limit of normal (ULN), liver enzymes (ALT and ALP) <5 × ULN, Cr <1.5 × ULN. Patients with any serious intercurrent medical illnesses were excluded, as were pregnant or lactating women. Women of child-bearing age were required to use effective contraception. The trial received approval from the local research ethics committee and all patients gave written informed consent to take part.

### Treatment of patients

Chemotherapy was administered on day 1 of a 21-day cycle. Cohorts of three patients were treated with a fixed dose of 5 g m^−2^ treosulfan (Medac, UK), preceded by escalating doses of gemcitabine (Lilly Oncology, UK), commencing at a dose of 0.5 g.m^2^ and increasing in increments of 0.5 g m^−2^. Treosulfan was administered as a 1-h infusion and gemcitabine as a 30-min infusion. Prophylactic intravenous and oral antiemetics were routinely used. For the first treatment cycle only, patients were allocated on an alternate basis to receive either gemcitabine or treosulfan alone. For all subsequent cycles, the combination regimen was given.

Physical examination, vital signs, toxicity evaluation (using the National Cancer Institute Common Toxicity Criteria version 2) and routine laboratory studies (FBC, biochemical profile, LDH) were undertaken prior to each cycle of treatment. Measurable disease was assessed every three cycles. Standard WHO criteria were used to assess tumour response. Complete response (CR) was defined as complete disappearance of all known disease determined by two observations not less than 4 weeks apart. Partial response (PR) was defined as a decrease of 50% or more in the sum of the products of the two maximum perpendicular diameters of assessable disease for at least 4 weeks, with no appearance of new lesions or progression of any lesion. Stable disease (SD) was defined as a less than 50% decrease or a less than 25% increase in the sum of the products of the two maximum perpendicular diameters of assessable disease. Progressive disease (PD) was defined as a 25% or more increase in the sum of the products of the two maximum perpendicular diameters of assessable disease or the development of any new lesions.

Dose-limiting toxicity (DLT) was predefined as any grade 3 or more toxicity (excluding alopecia, nausea and vomiting) experienced during the first cycle of combination therapy administered to any individual patient. When one patient experienced DLT, a total of six patients were treated at this dose level. The maximum tolerated dose (MTD) was defined if at least two out of six patients experience DLT. A further six patients were to be recruited at the dose level below the MTD to define the maximum recommended dose (MRD) for further study. The MRD was defined by a dose level that produced manageable and reversible toxicity.

### Drug-induced DNA damage

After cycles one and two, peripheral blood lymphocytes (PBLs) were collected from all patients at 0 h (pretreatment), 4, 24 and 48 h post-treatment. Peripheral blood lymphocytes were prepared on each occasion from 8 ml whole blood collected directly into a Vacutainer® CPT™ tube. Each blood sample was centrifuged at room temperature (1500 **g**) for 20 min. The white cell layer was collected and cold RPMI 1640 medium containing 10% foetal calf serum (FCS) and 2 mM L-glutamine was added. The sample was centrifuged at 4°C (200 **g**), for 5 min. The cells were resuspended in 2 ml RPMI 1640 medium containing 10% FCS, 2 mM L-glutamine and 10% dimethylsulphoxide and frozen as three aliquots at −70°C.

The single-cell gel electrophoresis (Comet) assay was used to detect and quantitate chemotherapy-induced DNA damage in patients' PBLs. Details of the Comet assay are described elsewhere (Spanswick *et al*, 1999). All procedures were carried out on ice and in subdued lighting. All chemicals used were obtained from Sigma Chemical Co. (Poole, UK) unless otherwise stated. Once thawed, each PBL sample was diluted to give a final concentration of 2.5 × 10^4^ ml^−1^. Each sample was then divided in two aliquots, irradiated and unirradiated. Immediately before analysis, the appropriate PBL samples were irradiated (10 Gy) in order to deliver a fixed number of random DNA strand breaks. After embedding cells in 1% low gelling temperature agarose on a precoated microscope slide, the cells were lysed for 1 h in lysis buffer (100 mM disodium EDTA, 2.5 M NaCl, 10 mM Tris-HCl pH 10.5) containing 1% Triton X-100 added immediately before analysis, and then washed every 15 min in distilled water for 1 h. Slides were then incubated in alkali buffer (50 mM NaOH, 1 mM disodium EDTA, pH12.5) for 45 min followed by electrophoresis in the same buffer for 25 min at 18 V (0.6 V cm^−1^), 250 mA. The slides were finally rinsed in neutralising buffer (0.5 M Tris-HCl, pH 7.5), and then in saline.

After drying, the slides were stained with propidium iodide (2.5 *μ*g ml^−1^) for 30 min, then rinsed in distilled water. Images were visualised using a NIKON inverted microscope with high-pressure mercury light source, 510–560 nm excitation filter and 590 nm barrier filter at × 20 magnification. Images were captured using an online CCD camera and analysed using Komet Analysis software version 4.0 (Kinetic Imaging, Liverpool, UK). For each duplicate slide, 25 cells were analysed. The tail moment for each image was calculated using the Komet Analysis software as the product of the percentage DNA in the comet tail and the distance between the means of the head and tail distributions, based on the definition of Olive *et al* (1990). In patients receiving single agent treosulfan, crosslinking was expressed as percentage decrease in tail moment compared to irradiated controls calculated by the formula: 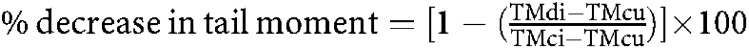


In patients receiving treosulfan and gemcitabine in combination, crosslinking was expressed as percentage decrease in tail moment compared to irradiated controls calculated by the formula below. This formula was used to compensate for the additional single-strand breaks induced by gemcitabine in addition to those produced by the irradiation step. 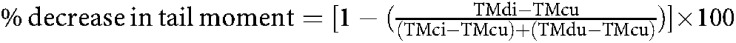 whereTMdi=tail moment of drug-treated irradiated sample; Tmdu=tail moment of drug-treated unirradiated sample; TMcu=tail moment of untreated, unirradiated control; TMci=tail moment of untreated, irradiated control.

## RESULTS

From August 29 2000 until November 13 2002, 27 advanced melanoma patients were enrolled, of whom 22 (81%) had cutaneous and five (19%) had uveal primaries. The median age was 50 (range 23–73) years. The majority of patients had metastases involving the liver (Table 1). In all, 10 patients (nine cutaneous, one uveal) had received prior chemotherapy with dacarbazine. A total of 17 (63%) patients had ECOG performances statue of zero at trial entry, the rest had an ECOG performance status of 1.

### Treatment administered and toxicity

All patients were assessable for toxicity (Table 2). The median number of chemotherapy cycles received was 5 (range 1–15). Dose-limiting toxicity was reached at 3.0 g m^−2^ gemcitabine, when two of six patients experienced grade III myelosupression comprising both neutropenia and anaemia. At the lower gemcitabine dose of 2.5 g m^−2^, only one episode of grade III neutropenia occurred. There were no episodes of febrile neutropenia. Other common toxicities considered to be probably or definitely related to chemotherapy administration were nausea and vomiting, fatigue, skin rash and constipation. At the start of the study, prophylactic domperidone or metoclopramide was routinely prescribed. However, at the higher gemcitabine doses of 2.5 g m^−2^ and above, use of a 5HT3 antagonist with or without dexamethasone proved to be more effective in controlling symptoms. A variety of musculoskelatal and abdominal pains were reported by patients treated in this study. Three patients treated between gemcitabine dose levels 1.5 and 3 g m^−2^ experienced severe abdominal pain, which could not be attributed to any obvious cause other than treatment. The known toxicities of gemcitabine, pulmonary toxicity, influenza-like symptoms and peripheral oedema, were closely monitored with dose –escalation; however, there did not appear to be an excess of events at the higher dose levels. The recommended dose for phase II evaluation associated with good patient tolerance was 2.5 g m^−2^ gemcitabine, combined with 5.0 g m^−2^ treosulfan.

### Response to treatment

A total of 26 patients were evaluable for response. No objective CRs or PRs were documented, using WHO criteria. Best responses were as follows: two (8%) minor responses (both uveal melanoma), 13 (46%) SD (10 cutaneous, two uveal) and 12 (46%) PD (10 cutaneous, one uveal). Overall disease control was therefore 54%. Median survival was 36 weeks (range 5–121), with one patient lost to follow-up. Median overall time to progression was 14 weeks (range 3–74). More detailed outcome data for the uveal melanoma patients is given in Table 3. The two minor responses occurred with doses of gemcitabine of 0.5 and 3.0 g m^−2^ in patients with extensive liver metastases. Median survival and time to progression for the uveal melanoma patients were 53 weeks (range 20–103) and 27 weeks (range 7–38), respectively.

### Comet assays

Comet assays were performed on samples from eight patients (Table 4) who received doses of gemcitabine ranging between 1.5 and 3.0 g m^−2^. Paired sets of samples (taken after cycle 1 administration of single agent and after cycle 2 administration of the combination regimen) were available for three patients, all of whom received gemcitabine alone as the first treatment cycle at a dose of either 2.5 or 3.0 g m^−2^.

**Figure 1:**
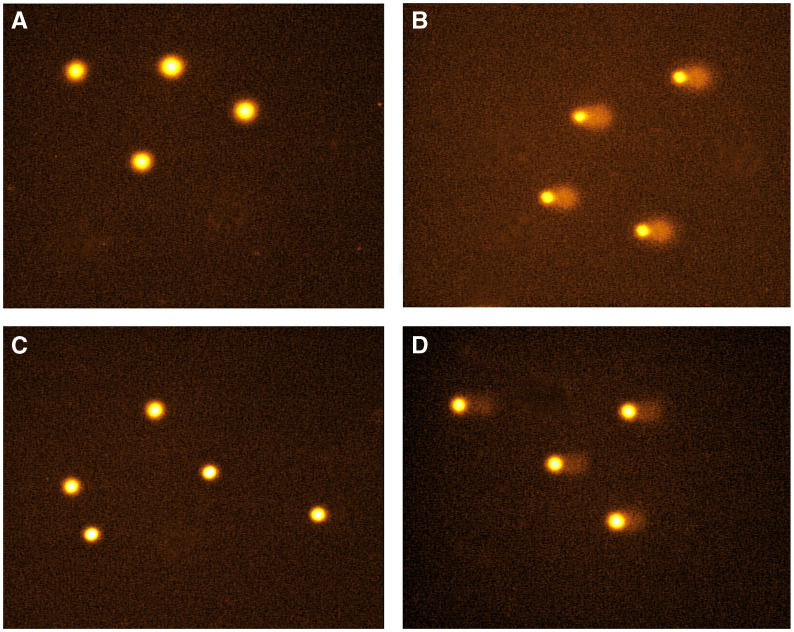
Typical comet images: (**A**) unirradiated PBLs taken prior to drug treatment, (**B**) irradiated PBLs taken prior to drug treatment, (**C**) unirradiated PBLs taken after treosulfan treatment and (**D**) irradiated PBLs taken after treosulfan treatment.

Typical comet images are shown in Figure 1. In control predose, unirradiated PBLs, no DNA damage was detected and the high molecular weight supercoiled DNA remained intact as shown in Figure 1A. Following irradiation of cells with 10 Gy to introduce a fixed level of random DNA single-strand breaks, the resulting shorter fragments of DNA migrated from the bulk of the DNA during electrophoresis to produce the typical comet images (Figure 1B). The extent of DNA damage was quantitated by image analysis to produce a tail moment, defined as the product of the percentage DNA in the comet tail and the distance between the means of the head and tail distributions, based on the definition of Olive *et al* (1990). In patients receiving treosulfan, both single agent and combination, the tail moment was used to calculate the percentage decrease in tail moment using the formula previously described. Therefore, the greater the percentage decrease the greater the level of DNA interstrand crosslinking. Following treosulfan treatment, no drug-induced single-strand breakage was detected in unirradiated cells (Figure 1C) and these cells showed similar profiles to the nondrug treated controls (Figure 1A). When the PBLs exposed to treosulfan were irradiated (Figure 1D), comet tails were visible but with decreased length and intensity compared to irradiated controls. The comet heads were larger and of greater intensity compared to the nondrug-treated irradiated control cells (Figure 1B) due to the retention of DNA by the treosulfan-induced interstrand crosslinks. The decrease in comet tail moment compared to nondrug-treated irradiated control was used to quantitate the level of DNA interstrand crosslinking. In contrast, following treatment with gemcitabine, single-strand breaks were observed.

**Figure 2:**
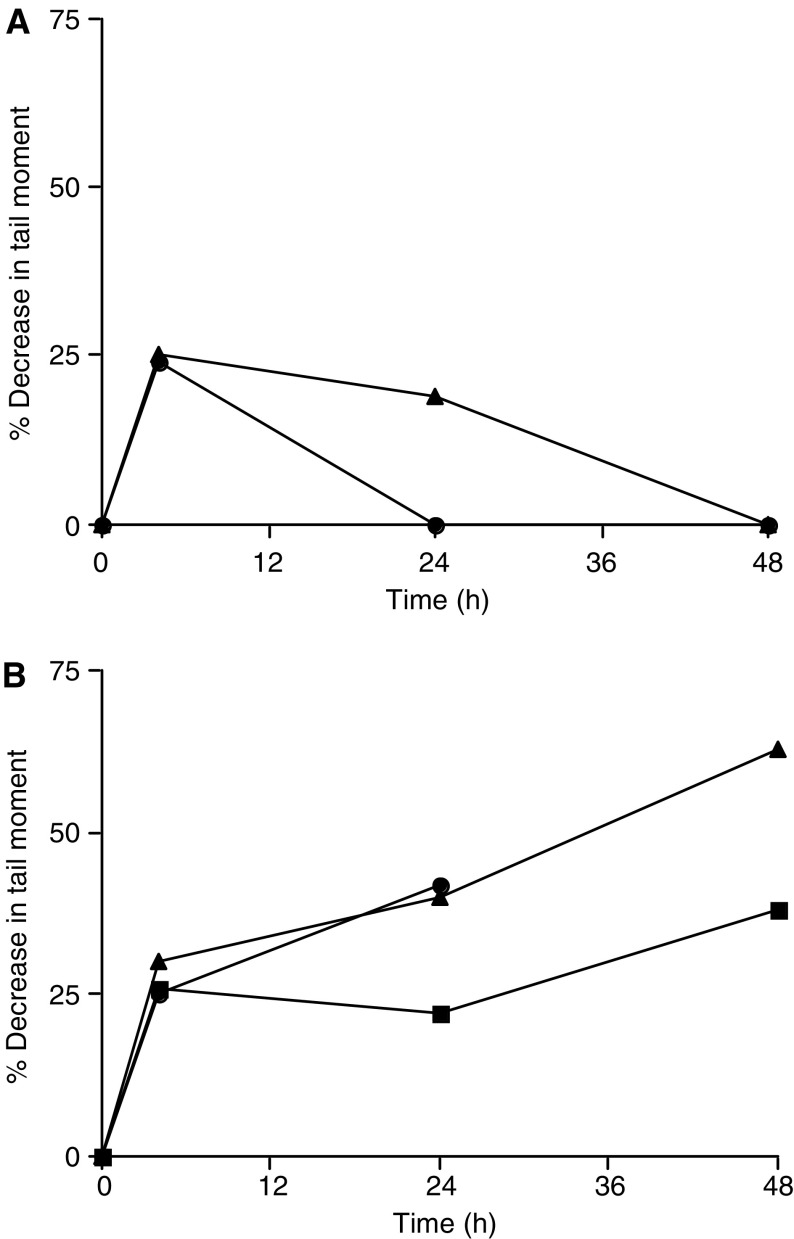
(**A**) Formation and repair of DNA interstrand crosslinks in lymphocytes from patients receiving treosulfan alone (patients 5 and 6). Results are expressed as percentage decrease in tail moment. (**B**) Persistence of treosulfan-induced DNA interstrand crosslinks following administration in combination with gemcitabine (patients 2, 3 and 8). Results are expressed as percentage decrease in tail moment.

Gemcitabine-induced DNA single-strand breaks were detected in PBLs following gemcitabine adminstration in the four patients treated (Patient numbers 1, 2, 3 and 8 in Table 4). They formed rapidly within 4 h post-treatment and were repaired by 24 h (data not shown). DNA interstrand crosslinks induced by treosulfan alone were detectable at 4 h in the two patients studied (Patient numbers 5 and 6 in Table 4) and were fully repaired within 48 and 24 h, respectively (Figure 2A). In three patients studied after receiving gemcitabine and treosulfan in combination (Patient numbers 2, 3 and 8 in Table 4), treosulfan-induced DNA interstrand crosslinks were detected at 4 h at equivalent levels to those seen in patients receiving treosulfan alone. However, increased levels of treosulfan-induced DNA interstrand crosslinks were detectable at 24 and 48 h (Figure 2B) and no repair was observed. These findings support the original hypothesis that gemcitabine may affect the persistance of treosulfan-induced DNA interstrand crosslinks.

## DISCUSSION

Gemcitabine combined with treosulfan is a novel combination chemotherapy regimen with preliminary preclinical and clinical evidence of activity in a variety of tumour types. The most promising results appear to be in uveal melanoma. The first phase I trial with this regimen combined gemcitabine and treosulfan on days 1 and 8 of a 28-day cycle in patients with various advanced solid tumours (Szelenyi *et al*, 1999). In this study, the dose of gemcitabine was fixed at 1 g m^−2^. The dose of treosulfan was then escalated from 2.5 g m^−2^ at 500 mg m^−2^ increments, and the recommended dose of treosulfan to be used in this combination schedule was 3.5 g m^−2^. The MTD was defined on conventional toxicity criteria, with thrombocytopaenia and neutropaenia being dose limiting. Of the original 20 patients enrolled in that study, two PRs (one renal, one ovary) and five (three uveal melanoma, two colorectal) minor responses were observed. This study was extended to include 33 patients with metastatic uveal melanoma. Although only one further partial response was observed, there were trends in improved survival at the highest treosulfan dose level (Schmittel *et al*, 2004).

A retrospective review of 14 patients with metastatic uveal melanoma treated in seven centres across Europe with four different schedules of gemcitabine (0.5–1 g m^−2^) combined with treosulfan (3.5–5 g m^−2^) given either once on a 21-day schedule, or on days 1 and 8 of a 28-day schedule, was recently reported (Pfohler *et al*, 2003). The objective response rate was 29% (one CR and three PRs), progression-free survival was 28 weeks, median overall survival was 61 weeks and 1-year survival rate was 80%. All of the schedules used were well tolerated, the most common side effects being neutropaenia and thrombocytopaenia.

The optimal schedule of gemcitabine combined with treosulfan has not yet been defined and the pharmacological basis for synergism existing between these two agents has not previously been ascertained. We hypothesised that the main cytotoxic event with the combination gemtreo regimen would be the formation of DNA interstrand crosslinks induced by treosulfan-derived products, which persist in the presence of gemcitabine. Since treosulfan is conventionally administered at doses of 5–7 g m^−2^ once every 21 days, in this phase I trial, we fixed the dose of treosulfan at 5 g m^−2^, escalated the dose of gemcitabine and measured the amount of DNA damage induced by each drug alone and by the combination regimen. Clinically, this combination regimen administered on a 3 weekly basis was well tolerated, with DLT being neutropaenia and anaemia at the gemcitabine dose level of 3.0 g m^−2^. No thrombocytopaenia was recorded. Other significant toxicities were fatigue and nausea, with sickness more effectively controlled by using prophylactic 5HT3 antagonist antiemetics. The recommended regimen for phase II evaluation associated with good patient tolerability is 2.5 g m^−2^ gemcitabine, combined with 5.0 g m^−2^ treosulfan, administered once every 3 weeks.

DNA damage induce by both drugs given alone or in combination was detected and quantitated using the single-cell gel electrophoresis (Comet) assay. The Comet assay allows measurement of DNA damage at a single-cell level. It has been modified to allow the sensitive detection and quantitation of DNA interstrand crosslinking and can be applied to both preclinical and clinical situations (Hartley *et al*, 1999a, 1999b, 2004; Spanswick *et al*, 2002). Comet assay results could only be obtained from a limited number of patients treated in this clinical study. Even so, single-strand DNA breaks were consistently detected following gemcitabine alone at 4 h and were fully repaired by 24 h (data not shown). Interstrand DNA crosslinks induced by treosulfan alone were detectable at 4 h and were fully repaired by 48 h. Following combination chemotherapy, treosulfan-induced DNA crosslinks persisted, being detectable at high levels 48 h post-treatment. These Comet assay results support the general hypothesis that gemcitabine may affect the persistance of treosulfan-induced DNA interstrand crosslinks.

The optimal dose of gemcitabine recommended from this study to be used in the gemtreo combination regimen is potentially at odds with current understanding of the intracellular metabolism of gemcitabine, which is considered to be saturated at an optimal fixed dose rate of 10 mg m^−2^ min^−1^ (Tempero *et al*, 2003). These comet assay data were insufficient to determine the existence or otherwise of a dose effect on DNA damage induced by increasing gemcitabine doses used in this study.

In terms of clinical efficacy, although no objective responses were seen, two of five uveal melanoma patients entered in this trial, both with extensive liver metastases, experienced a minor response and these occurred in patients treated at the lowest and highest gemcitabine dose levels. The limited number of uveal melanoma patients treated in this trial prohibits any firm conclusions regarding the possibility of a gemcitabine dose response. However, these findings would certainly support previously published clinical data suggesting that the gemtreo regimen is an active regimen in the treatment of metastatic uveal melanoma.

## Figures and Tables

**Table 1:** Patient characteristics

**Characteristic**	**No. of patients**	**%**
Total no of patients	27	
*Sex*		
Male	14	52
Female	13	48
		
*Age*		
Median	50	
Range	23–73	
		
*ECOG performance status*		
0	17	63
1	10	37
		
*Melanoma primary site*		
Cutaneous	22	81
Uveal	5	19
		
*Sites of metastasis*		
M1a	3	11
M1b	7	26
M1c	17	63
		
*Prior chemotherapy*		
Cutaneous	9	33
Uveal	1	4

**Table 2:** Toxicities identified as either possibly, probably or very likely to be related to gemtreo combination chemotherapy

	**Gemcitabine dose level (g m^−2^)**											
	**0.5**		**1.0**		**1.5**		**2.0**		**2.5**		**3.0**	
No. of pts treated	3		3		3		3		8		7	
						
Total no. of treatment cycles	22		37		24		13		42		39	
						
Toxicity grade	1/2	3	1/2	3	1/2	3	1/2	3	1/2	3	1/2	3
												
Toxicity												
												
*Haematological toxicity*												
Neutropenia	0	0	0	0	0	0	0	2	1	2	2	2
Anaemia	0	0	0	0	0	0	0	0	8	0	2	4
Thrombocytopaenia	0	0	0	0	0	0	0	0	0	1	0	0
												
*Nonhaematological toxicity*												
N/V	0	0	1	0	1	0	1	1	26	1	15	2
Anorexia	5	0	3	0	5	0	2	0	12	0	11	2
Diarrhoea	0	0	0	0	0	0	0	0	1	0	0	0
Constipation	0	0	0	0	0	0	0	0	9	1	5	0
Fatigue	5	0	3	0	15	0	3	0	26	2	13	2
Skin rash 0	0	0	0	0	0	0	0	6	0	15	0	
Oedema	0	0	1	0	0	0	0	0	1	0	0	1
Respiratory	1	0	3	0	1	0	0	0	3	0	2	0
Neurological	0	0	1	0	0	0	3	1	7	0	2	0
M/S	0	0	1	0	3	0	6	0	17	0	6	0
Abdominal pain	1	0	0	0	3	1	2	0	3	1	3	1
Alopecia	0	0	0	0	0	0	0	0	3	0	7	1
CVS	0	0	1	0	0	0	0	0	0	0	0	0
Elevated LFTs	0	0	0	0	0	0	0	0	0	0	2	0
Other	1	0	3	0	0	0	1	1	6	0	9	0

N/V=nausea and vomiting; CVS=cardiovascular symptoms; M/S=musculoskeletal symptoms; LFTs=liver function tests; pts=patients.

**Table 3:** Clinical outcomes for the uveal melanoma patients

**Trial Pt ID.**	**Gemcitabine dose (g m^−2^)**	**Sites of metastasis**	**First /second-line therapy**	**No. of cycles**	**Best response**	**TTP (weeks)**	**Survival (weeks)**
03	0.5	Liver	First	12	MR	38	53
04	1.0	Lungs	First	3	Progression	7	20
10	2.0	Liver, skin, bone	First	6	Stable	14	53
19	3.0	Liver	First	9	Stable	30	88
21	3.0	Liver	Second	9	MR	27	103

TTP=time to progression; MR=minor response.

**Table 4:** Details of patient samples on which Comet assays were successfully performed

**Patient number**	**Primary tumour**	**Gemcitabine dose (g m^−2^)**	**Cycle 1 PBLs: Treosulfan alone**	**Cycle 1 PBLs: Gemcitabine alone**	**Cycle 2 PBLs: Gem/Treo combination**
1	Skin	1.5	—	Yes	No
2	Skin	2.5	—	Yes	Yes
3	Skin	2.5	—	Yes	Yes
4	Skin	2.5	—	—	Yes
5	Skin	3.0	Yes	—	No
6	Skin	3.0	Yes	—	No
7	Skin	3.0	No	—	Yes
8	Uveal	3.0	—	Yes	Yes
